# Development of a standardized method for contouring the larynx and its substructures

**DOI:** 10.1186/s13014-014-0285-4

**Published:** 2014-12-11

**Authors:** Mehee Choi, Tamer Refaat, Malisa S Lester, Ian Bacchus, Alfred W Rademaker, Bharat B Mittal

**Affiliations:** Department of Radiation Oncology, Northwestern University, Robert H. Lurie Comprehensive Cancer Center, 251 E Huron, LC-178, Chicago, IL 60611 USA; Department of Radiology, Northwestern University, Robert H. Lurie Comprehensive Cancer Center, Chicago, Illinois USA; Preventive Medicine, Northwestern University, Robert H. Lurie Comprehensive Cancer Center, Chicago, Illinois USA; Department of Radiation Oncology, Stritch School of Medicine Loyola University Chicago, Cardinal Bernardin Cancer Center, Chicago, Illinois USA; Department of Clinical Oncology, Faculty of medicine, Alexandria University, Alexandria, Egypt

**Keywords:** Head-and-neck cancer, Larynx anatomy, Swallowing dysfunction, Intensity-modulated radiotherapy, Organs at risk

## Abstract

**Objectives:**

Limiting radiation dose to the larynx can diminish effects of laryngeal dysfunction. However, no clear guidelines exist for defining the larynx and its substructures consistently on cross-sectional imaging. This study presents computed tomography (CT)- and magnetic resonance imaging (MRI)-based guidelines for contouring laryngeal organs-at-risk (OARs).

**Materials and Methods:**

Standardized guidelines for delineating laryngeal OARs were devised and used to delineate on CT and MRI for head-and-neck cancer patients. Volumetric comparisons were performed to evaluate consistency and reproducibility of guideline-based contours.

**Results:**

For the initial 5 patients the mean CT and MRI based larynx volume did not differ significantly between imaging modalities; 34.39 ± 9.85 vs. 35.01 ± 9.47 (p = .09). There was no statistical difference between the CT based mean laryngeal volume in the subsequent 44 patients compared to the initial 5 patients outlined on CT and the MRI scan (p = 0.53 and 0.62). The OAR volume for laryngeal substructures were not statistically different among patients or between imaging modalities. Once established, the guidelines were easy to follow.

**Conclusion:**

The guidelines developed provide a precise method for delineating laryngeal OARs. These guidelines need to be validated and clinical significance of outlining laryngeal substructures and dose-volume constraints should be investigated before routine implementation in clinic practice.

## Background

The larynx plays an important role in speech and swallowing. Progressive laryngeal edema and fibrosis following radiotherapy for head and neck cancer can lead to long-term problems with phonation and swallowing and significantly compromise quality of life in cancer survivors [1,2]. The incidence of swallowing dysfunction significantly increases with intensified regimens, such as the addition of chemotherapy to radiotherapy [3-5]. Several studies have shown that reduced radiation dose to the larynx can diminish the effects of laryngeal dysfunction [6-9]. It remains unclear which substructures of the larynx, when irradiated, are most associated with swallowing dysfunction*.*

With the advent of technologies such as intensity–modulated radiotherapy (IMRT), it is possible to selectively spare dose to the larynx and its substructures as organs at risk (OARs), thereby reducing the risk of speech and swallowing dysfunction [1]. This has prompted the Radiation Therapy Oncology Group (RTOG) to require larynx contours with dose constraints of mean dose ranging from 36 to 45 Gy on many recent protocols. Larynx-sparing radiotherapy requires that radiation oncologists follow a common methodology for contouring the larynx and its substructures. However, to date there has been no validated standardized approach for contouring the larynx and its substructures on axial computed tomography (CT) scans or magnetic resonance imaging (MRI) scans used for radiotherapy treatment planning (Table 1). The purpose of this study was to devise standardized step-by-step guidelines for contouring the larynx and its substructures for use in IMRT plans and radiation induced speech and swallowing dysfunction research.

**Table 1:** **Current RTOG head and neck protocols requiring larynx contours**

**Protocol**	**Constraint**	**Contouring instructions**
**RTOG 1016:** Phase III trial of radiotherapy plus cetuximab vs chemoradiotherapy in HPV-positive oropharynx cancer	Reduce the dose as much as possible	GSL: "triangular prism-shaped" volume that begins just inferior to the hyoid bone and extends to the cricoid cartilage inferiorly and extends from the anterior commissure to include the arytenoids. This includes the infrahyoid but not the suprahyoid epiglottis
	Glottic larynx mean dose ≤ 20 Gy (2Gy/fx)	
**RTOG 1008:** Phase II study of adjuvant concurrent radiation and chemotherapy vs radiation alone in resected high-risk malignant salivary gland tumors	Reduce the dose as much as possible Larynx mean dose <35 Gy whenever feasible (2 Gy/fx)	Same as RTOG 1016
**RTOG 0920:** Phase III study of postoperative radiation therapy +/− cetuximab for locally advanced resected head and neck cancer	Reduce the dose as much as possible Larynx mean dose <45 Gy whenever feasible (2 Gy/fx)	Same as RTOG 1016
**RTOG 0912:** Phase II study of concurrent intensity-modulated radiation therapy, paclitaxel, and pazopanib/placebo, for the treatment of anaplastic thyroid cancer	Glottic larynx mean dose <60 Gy (2Gy/fx)	None provided

*Abbreviations:* RTOG = Radiation Therapy Oncology Group; fx = fractions; GSL = glottic/supraglottic larynx.

## Methods

This study was part of an Institutional Review Board (IRB)-approved project. Anatomic textbooks and radiologic data were reviewed for descriptions of the larynx and its substructures [10,11]. A board-certified neuroradiologist assisted with identification of the larynx and laryngeal substructures as well as adjacent structures including the oral cavity, oropharynx, pharyngeal constrictors, and hypopharynx using axial CT. The following step-by-step technique for contouring the larynx and its substructures on axial CT was devised. Similar guidelines can be used to contour the larynx on T1-weighted MRI scans.

The study was approved by Northwestern University institutional review board.

Use a bone window for the following:

1. Identify and contour the thyroid cartilage. The two ala of the thyroid cartilage fuse anteriorly to form a V-shaped shield. The superior and inferior cornua project from the posterior free edges of the thyroid cartilage.
2. Identify and contour the cricoid cartilage. The cricoid cartilage forms a complete ring to form the base and back of the larynx; it forms a narrow rim anteriorly and a broad lamina posteriorly. Superiorly, it begins just below the arytenoid cartilages. Inferiorly, it ends just above the first tracheal ring.
3. Identify and contour the arytenoid cartilages. This pair of pyramid-shaped cartilages sits directly on the posterior rim of the cricoid cartilage and posteromedial to the thyroid cartilage.
4. Identify and contour the glottic larynx, which sits on the same axial plane as the inferior edge of the arytenoid cartilages. Anteriorly and laterally, the glottic larynx is bound by the postero-medial edge of the thyroid cartilage. Posteriorly, it is bound by the anterior edge of the arytenoid cartilages.
5. Identify and contour the subglottic larynx. This area is composed of the airspace and mucosa housed by the cricoid cartilage. Superiorly, it begins at the slice below the glottic larynx. Inferiorly, it ends at the same level as the most inferior slice of the cricoid cartilage.

Use a soft tissue window with good definition between muscle and fat densities for the following:

6. Identify and contour the suprahyoid portion of the epiglottis, a leaf-like cartilage that hovers over the glottic inlet at and above the level of the hyoid bone. Superiorly, it sits in air within the inferior oropharynx and extends inferiorly to the level of the bottom slice of the hyoid bone. Note that the epiglottis forms the anterior wall of the laryngeal vestibule. Typically, a clear fat plane can be seen wrapping antero-laterally around the epiglottis and should not be included.
7. Identify and contour the infrahyoid epiglottis. This structure begins below the inferior aspect of the suprahyoid epiglottis. Inferiorly, the epiglottis forms a narrow stem that attaches to the posterior surface of the angle of the thyroid cartilage and ends just above the glottic larynx.
8. Because they are difficult to differentiate from one another without direct visualization, identify and contour the aryepiglottic folds and false vocal folds as a single structure. Superiorly, the structure begins at the superior aspect of the valleculae, forming the lateral walls of this structure. Inferiorly, the structure forms the lateral wall of the supraglottic larynx and medial wall of the pyriform sinuses.
9. Create the epiglottis OAR by combining the contours of the suprahyoid epiglottis and infrahyoid epiglottis contours.
10. Create the supraglottic larynx OAR by combining the contours for the epiglottis, arytenoids, and the antero-medial wall of the aryepiglottic folds and false vocal folds. The postero-lateral wall of the aryepiglottic folds forms the medial wall of the pyriform sinuses and is part of the hypopharynx.
11. Create the larynx OAR by combining the supraglottic larynx, glottic larynx, subglottic larynx, thyroid cartilage, and cricoid cartilage contours.

Using these guidelines, the larynx OARs were contoured on the radiotherapy treatment-planning CT scans using the Pinnacle treatment-planning system (ADAC Philips Pinnacle 3 version 8.6™) for five consecutive patients who were undergoing definitive chemoradiation for locally advanced head-and-neck cancer of a non-larynx primary.

**Figure 1:**
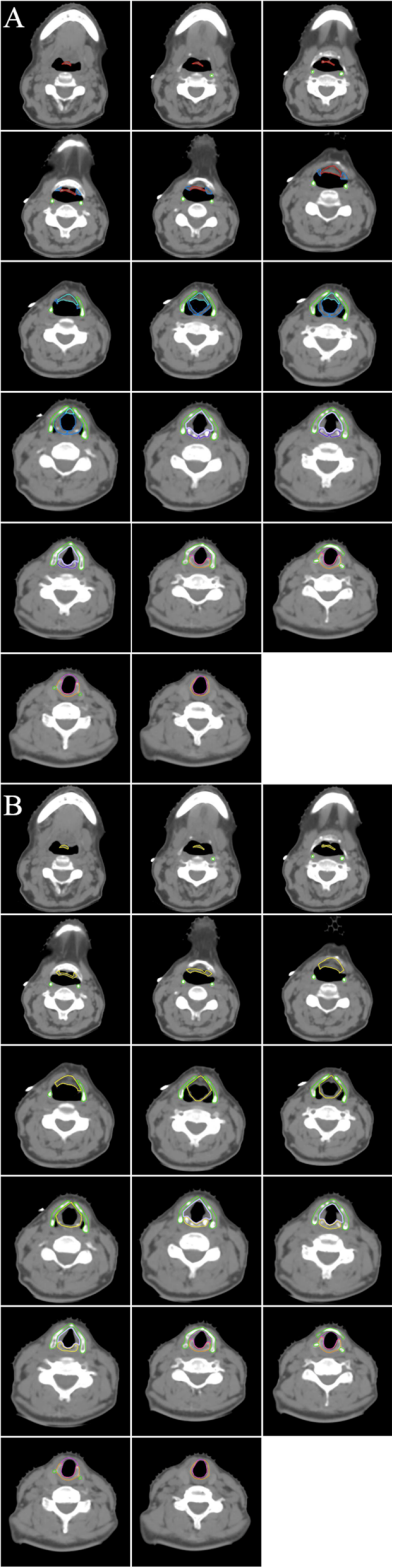
**Atlas of the larynx and its substructures on consecutive axial computed tomography (CT) slices: the thyroid cartilage is depicted in green, the cricoid cartilage in orange, the arytenoid cartilages in purple, the suprahyoid epiglottis in red, the infrahyoid epiglottis in cyan, the aryepiglottic fold/false vocal folds in blue, the supraglottic larynx in yellow, the glottic larynx in lavender, and the subglottic larynx in magenta. (A)** Individual substructures of the larynx. **(B)** Major divisions of the larynx.

**Figure 2:**
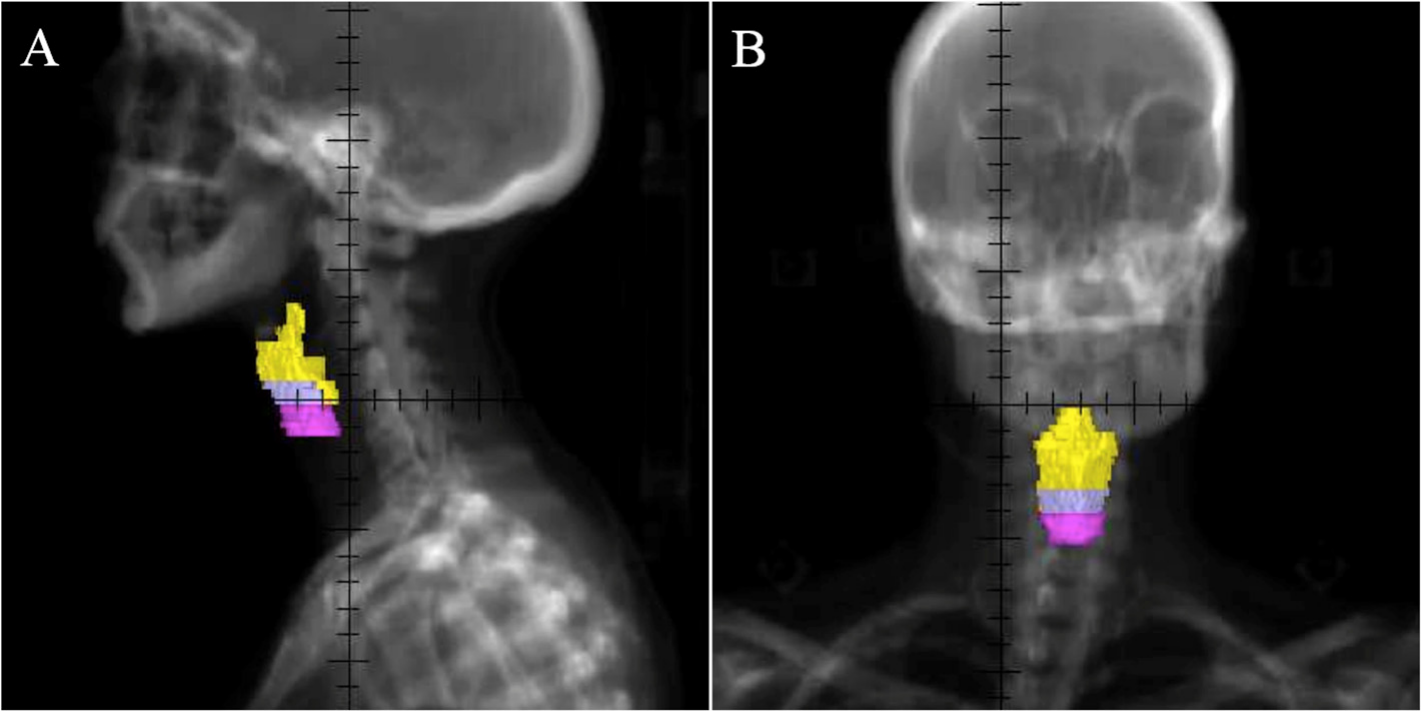
**Digitally reconstructed radiographs of the major divisions of the larynx generated from contours shown in Figure** **1** **B.** This view provides visual approximation of the supraglottic larynx, glottic larynx, and subglottic larynx volumes generated from our guidelines. The supraglottic larynx is depicted in yellow, the glottic larynx in lavender, and the subglottic larynx in magenta. **(A)** Reconstructed sagittal view. **(B)** Reconstructed coronal view.

The OARs were delineated by one radiation oncologist and reviewed and adjusted when considered appropriate by one other radiation oncologist and a neuroradiologist. These assessments resulted in a consensus determination of the OARs. Examples of the OARs are shown in Figures 1 and 2.

**Figure 3:**
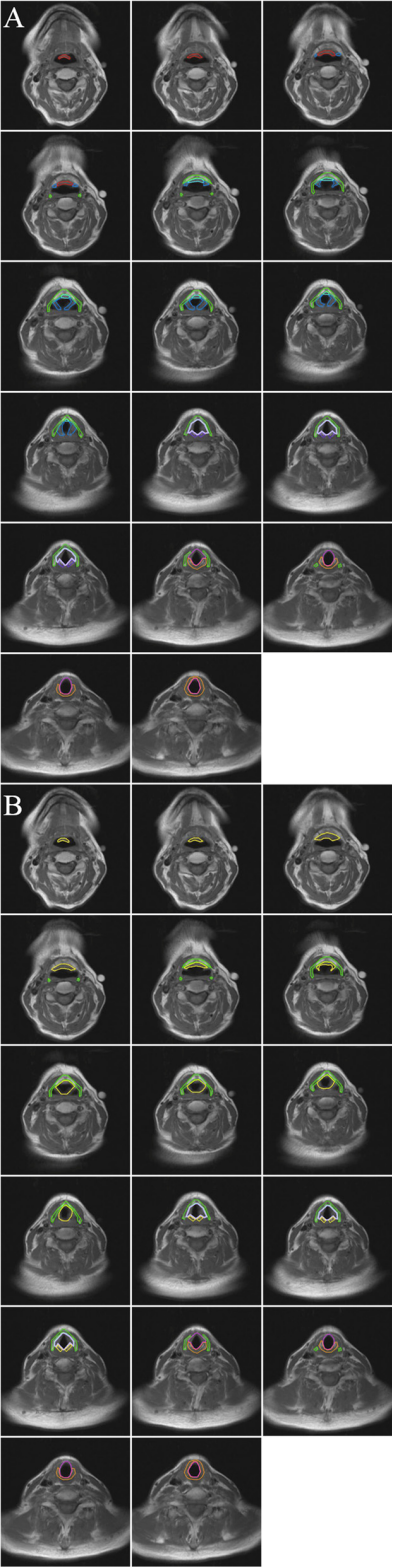
**Atlas of the larynx and its substructures on consecutive axial magnetic resonance imaging (MRI) slices, T1-weighted, pre-contrast sequence: the thyroid cartilage is depicted in green, the cricoid cartilage in orange, the arytenoid cartilages in purple, the suprahyoid epiglottis in red, the infrahyoid epiglottis in cyan, the aryepiglottic fold/false vocal folds in blue, the supraglottic larynx in yellow, the glottic larynx in lavender, and the subglottic larynx in magenta. (A)** Individual substructures of the larynx. **(B)** Major divisions of the larynx.

MRI provides visualization of soft-tissue planes superior to that seen on CT and is frequently used in head and neck cancers for tumor staging and determining surgical resectability [11]. As such, to validate the accuracy of the CT-based contouring guidelines, the larynx volumes were drawn independently by one radiation oncologist on axial MRI (T1-weighted, pre-contrast sequence) for the same five patients using the CT-based contouring guidelines. These contours were reviewed and verified by a board-certified neuroradiologist resulting in a consensus contour. Examples of the OARs are shown in Figure 3. The volumes of the larynx and its substructures were compared for both CT and MRI. For comparison between CT and MRI contours, a two-sided paired t-test was performed for each structure, and *p* values <0.05 were considered significant.

Once internally agreed upon, the guidelines were used to delineate the larynx on radiotherapy treatment planning CT scans for an additional 44 patients treated with chemoradiation for head and neck cancer of a non-larynx primary. The contours were delineated by one radiation oncologist and reviewed by another radiation oncologist, resulting in consensus contours. Volumetric comparisons were made between the guideline-based CT contours for these 44 patients and the CT- and MRI-based contours for the initial five patients using a two-sided independent sample t-test for each structure, and *p* values <0.05 were considered significant.

## Results

A total of 49 patients with head and neck squamous cell carcinoma were included in this study. Forty patients (82%) were men and 9 patients (18%) were women. The primary tumor sites were oropharynx, unknown primary, nasopharynx, and hypopharynx for 37 (76%), 9 (18%), 2 (4%), and 1 (2%) patients, respectively. No patients had primary laryngeal cancer. Forty-four patients had stage IV disease, 4 had stage III disease, and 1 had stage II disease. Median age at diagnosis was 54 years (range, 30–74).

The larynx and its substructures were successfully created independently on CT and MRI datasets initially for five patients using the proposed larynx OAR guidelines. Table 2 shows the volumes of the OARs contoured on both MRI and CT. Differences in OAR volume (cubic centimeter) were not statistically different.

**Table 2:** **CT/MRI comparison of OAR volumes for five patients with locally advanced head and neck cancer**

**Organ at risk**	**CT (mean ± SD)**	**MRI (mean ± SD)**	***p*** **value**
Thyroid cartilage, cm^3^	8.51 ± 3.42	8.32 ± 3.07	0.50
Cricoid cartilage, cm^3^	3.89 ± 1.48	3.84 ± 1.49	0.82
Arytenoid cartilages, cm^3^	1.12 ± 0.28	1.12 ± 0.28	0.81
Suprahyoid epiglottis, cm^3^	1.77 ± 0.93	1.86 ± 0.72	0.58
Infrahyoid epiglottis, cm^3^	0.96 ± 0.80	0.84 ± 0.62	0.42
Epiglottis, cm^3^	2.73 ± 0.95	2.63 ± 0.87	0.17
Aryepiglottic folds/false vocal folds, cm^3^	7.03 ± 3.39	7.08 ± 3.47	0.58
Supraglottic larynx, cm^3^	12.83 ± 3.78	12.19 ± 4.71	0.23
Glottic larynx, cm^3^	3.14 ± 0.37	3.25 ± 0.49	0.33
Subglottic larynx, cm^3^	4.96 ± 1.52	5.15 ± 1.43	0.16
Larynx, cm^3^	34.39 ± 9.85	35.01 ± 9.47	0.09

*Abbreviations:* OAR = organ at risk; SD = standard deviation; CT = computed tomography-based volumes; MRI = magnetic resonance imaging-based volumes.

For the 44 additional patients contoured, the mean CT-based larynx volume was 37.2 ± 9.2 cm^3^. Mean volumes for the supraglottic larynx, glottic larynx, and subglottic larynx were 13.9 ± 3.7 cm^3^, 3.0 ± 0.7 cm^3^, and 5.6 ± 1.9 cm^3^, respectively. Comparison of these 45 CT-based contours with the five initial CT-based contours and MRI-based contours showed no significant difference in OAR volumes. Table 3 summarized the volumes for the complete set of OARs in the additional patients.

**Table 3:** **CT-based larynx OAR volumes for 44 additional patients with locally advanced head and neck cancer**

		***p*** **value**	
**Organ at risk**	**Mean ± SD**	**CT**_**44 pts**_ **–CT**_**5 pts**_ *	**CT**_**44 pts**_ **–MRI**_**5 pts**_ *
Thyroid cartilage, cm^3^	10.2 ± 3.11	0.26	0.21
Cricoid cartilage, cm^3^	3.83 ± 1.29	0.93	0.99
Arytenoid cartilages, cm^3^	1.00 ± 0.39	0.55	0.52
Suprahyoid epiglottis, cm^3^	1.94 ± 0.86	0.67	0.83
Infrahyoid epiglottis, cm^3^	0.96 ± 0.51	0.99	0.61
Epiglottis, cm^3^	2.91 ± 1.09	0.73	0.59
Aryepiglottic folds/false vocal folds, cm^3^	6.11 ± 1.72	0.32	0.29
Supraglottic larynx, cm^3^	13.87 ± 3.68	0.56	0.36
Glottic larynx, cm^3^	2.99 ± 0.70	0.64	0.44
Subglottic larynx, cm^3^	5.55 ± 1.94	0.52	0.66
Larynx, cm^3^	37.20 ± 9.20	0.53	0.62

*Abbreviations:* OAR = organ at risk; SD = standard deviation; CT = computed tomography-based volumes; MRI = magnetic resonance imaging-based volumes; pts = patients.

*Comparisons are between volumes obtained from 44 additional patients and five initial patients.

Since there is evidence in the literature that the larynx is larger in men than in women, we also decided to look at larynx volume by gender. Mean volumes for the CT-based larynx contours were significantly smaller for women than for men (*p* < 0.05). For women, mean volumes for the larynx, supraglottic larynx, glottic larynx, and subglottic larynx were 20.1 ± 3.0 cm^3^, 7.4 ± 1.6 cm^3^, 2.2 ± 0.5 cm^3^, and 2.9 ± 0.6 cm^3^, respectively. For men, mean volumes for the larynx, supraglottic larynx, glottic larynx, and subglottic larynx were 40.7 ± 4.9 cm^3^, 15.1 ± 2.3 cm^3^, 3.2 ± 0.6 cm^3^, and 6.1 ± 1.6 cm^3^, respectively.

## Discussion

In our study, we developed simple step-by-step CT-based guidelines for delineating the larynx and its substructures within radiation treatment plans for patients undergoing IMRT for head and neck cancer. This study provides initial validation that these contouring guidelines can be applied to radiotherapy planning for CT scans by comparing them to MRI contours. These guidelines can potentially serve as a research tool and can help reduce observer variability on OAR delineation, allowing for improved comparison and interpretation of dose–volume effects for these OARs from different studies.

Radiation–associated dysphagia is a common and often permanent late complication of radiotherapy to the head and neck. Only a limited number of studies have attempted to define the most important anatomic structures whose dose–volume parameters may have a major effect on swallowing. Candidate structures that have been associated with functional dysphagia endpoints have included the larynx, pharyngeal constrictors, and upper esophagus [6-9,12,13]. Delineation guidelines for the pharyngeal constrictors and esophagus exist and are commonly used in daily practice and clinical trials [7,12,14-16]. Other sets of proposed guidelines for the larynx have been put forward but are sparse; none provide clear, comprehensive guidelines for delineating the larynx in its entirety [7,12,14-16]. To our knowledge, delineation guidelines for contouring laryngeal substructures, as presented in this paper, do not exist.

It should be noted that imaging modalities other than CT, such as MRI, might improve visualization of the larynx and surrounding structures. MRI, with its superior soft-tissue contrast, can help to discriminate the laryngeal substructures from surrounding muscle and fat and can provide the best tumor visibility [10,11]. Therefore, the CT-based contouring guidelines developed here were also used to contour on axial MRI for five patients. The T1-weighted, pre-contrast sequence was selected because it generally has good anatomic detail, with fat as inherent contrast, and is less susceptible to artifact as compared to other sequences. Volumetric comparison showed the CT and MRI volumes to be comparable, suggesting that CT-based delineation is adequate for evaluation of these structures. The guidelines developed here could be used for contouring on MRI. This may be of interest as new radiotherapy treatment systems with online MR imaging are developed and gain wider use in the clinic [17].

To decrease radiation to organs at risk, it is essential to accurately contour the structures of interest. A number of investigators have reported on larynx–sparing IMRT techniques, such as junctioned IMRT and IMRT with modulated arcs, acknowledging that if the larynx is incorporated into the optimization process, larynx dose can be reduced significantly from a mean dose of approximately 50 Gy, typically found when laryngeal sparing is not attempted, to 25 to 40 Gy, while maintaining acceptable target coverage [18-22]. Delineation of the larynx and its substructures has not been specified in the majority of these studies.

However, some investigators express concern that reducing dose to the larynx in this way could compromise dose distribution elsewhere [23]. To address this issue in a meaningful way, accurate contouring and planning of the laryngeal OARs are critical [24]. Standardization of delineation protocols should help to improve such optimization of larynx-sparing radiation therapy in head and neck cancer.

When using these guidelines, it should be noted that differences exist among patients, and the delineation of individual variants should be addressed by the treating physicians. For example, our findings corroborate, using CT scan, the finding by Hollien, *et al.* who estimated that the size of the larynx is larger in men than in women using x-ray technology [25]. Furthermore, when the tumor alters the normal anatomy, delineation of the involved laryngeal substructures may be of limited clinical utility as they may have impaired functionality as a result of tumor invasion. Finally, imaging of the larynx can be challenging given its mobility and its proximity to other structures (*e.g.,* pharyngeal constrictors) that can cause motion artifact. As such, imaging acquisition should be optimized to minimize artifact from breathing and swallowing: the neck should be hyperextended to help reduce the frequency of swallowing, and the patient should be instructed to resist swallowing or coughing [11].

The contouring guidelines presented provide an easy tool for comprehensively delineating larynx and its substructures. Our study has limitations, as we did not assess inter-delineator variability or take laryngeal motion into account. However, these guidelines are a consensus opinion of an experienced head and neck radiation oncologist and a neuro-radiologist, based upon the literature review of laryngeal anatomy. It remains to be seen if any single or multiple laryngeal substructures play a preferential and significant role in speech and swallowing. Further validation within the context of a prospective clinical trial is required in order to assess if utilizing this contouring approach would result in lower incidence of treatment-induced adverse events; mainly hoarseness of voice, aspiration and dysphagia. Towards this end, our study can serve as a research tool in contouring and investigating dose-volume constraints of laryngeal substructures. The guidelines will promote consistency in contouring and reducing inter-observer variation, which has been shown to have a large impact on target and normal tissue delineation [26].

## Conclusions

We provide a precise and accurate method for delineating the larynx and its substructures on treatment-planning CT scans. These guidelines should be validated and can be used as a research tool to understand clinical significance of contouring laryngeal substructures and their importance in dose-optimization. The validated contouring guidelines will reduce inter-observer variability and lead to an improved understanding of dose-volume relationship of larynx and its substructures to consequent speech and swallowing dysfunction.

## Abbreviations

CT: Computed Tomography
IMRT: Intensity-Modulated Radiotherapy
IRB: Institutional Review Board
MRI: Magnetic Resonance Imaging
OARs: Organs at risk
RTOG: Radiation Therapy Oncology Group
